# Supplementary material for: Micronuclei-based model system reveals functional consequences of chromothripsis in human cells
Source: eLife. 2019 Nov 28;8:e50292. doi: 10.7554/eLife.50292 (PMC6910827; doi:10.7554/eLife.50292)
Supplement: Supplementary file 1. — The cell lines were created by microcell-mediated chromosome transfer. The % of cells with whole chromosome aneuploidy (WCA) shows the % of metaphase spreads that scored positive for the expected WCA. [file elife-50292-supp1.docx]

**Supplementary File 1 | Cell lines used in the study.**

The cell lines were created by microcell-mediated chromosome transfer. The % of cells with whole chromosome aneuploidy (WCA) shows the % of metaphase spreads that scored positive for the expected WCA.

|  | **HCT116-derived cell lines** | | | | | |
| --- | --- | --- | --- | --- | --- | --- |
|  | **Name** | **Chr.** | **H2B-GFP** | **% cells with WCA** | **Analysis** | **Comments** |
|  | HCT116 | - | - | 0 | SNP, WGS, CGH | AATC |
|  | HCT116 H2B-GFP | - | + | 0 | SNP, WGS,  CGH | Kuffer et al, 2013 |
| 1 | Htr3-11 | 3 | + | 92 | SNP, CGH | Passerini et al, 2016 |
| 2 | Htr3-13 | 3 | + | 85 | SNP | Passerini et al, 2016 |
| 3 | Htr3-14 | 3 | + | 92 | SNP | This work |
| 4 | Hte5-01 | 5 | - | 96 | SNP, WGS, CGH | From Minoru Koi (Stingele et al, 2012) |
| 5 | Hte5-04 | 5 | + | 83 | CGH | Stingele et al, 2012 |
| 6 | Htr5-06 | 5 | + | 92 | CGH | Stingele et al, 2012 |
| 7 | Htr5-11 | 5 | + | 100 | SNP,SMASH | This work |
| 8 | Htr5-12 | 5 | + | 92 | SNP,SMASH | This work |
| 9 | Htr5-13 | 5 | + | 100 | SNP,SMASH | This work |
| 10 | Hte5-14 | 5 | + | 92 | SNP,SMASH | This work, Tetrasomy |
| 11 | Htr5-15 | 5 | + | n/a | SMASH | This work |
| 12 | Htr5-16 | 5 | + | 97 | SNP,SMASH | This work |
| 13 | Htr5-17 | 5 | + | 83 | SNP,SMASH | This work |
| 14 | Htr5-18 | 5 | + | 73 | SNP,SMASH | This work  One extra chrom. + extra arm |
| 15 | Htr5-19 | 5 | + | 66 | SNP | This work  Only extra arm |
| 16 | Htr8-01 | 8 | + | 78 | SNP | Donnelly et al, 2014 |
| 17 | Htr8-02 | 8 | + | 70 | SNP | Donnelly et al, 2014 |
| 18 | Htr8-03 | 8 | + | 73 | SNP | Donnelly et al, 2014 |
| 19 | Htr8-04 | 8 | + | 86 | SNP | Donnelly et al, 2014 |
| 20 | Htr8-05 | 8 | + | 89 | SNP | Donnelly et al, 2014 |
| 21 | Htr8-06 | 8 | + | 75 | SNP | Donnelly et al, 2014 |
| 22 | Htr8-07 | 8 | + | 100 | SNP | Donnelly et al, 2014 |
| 23 | Htr8-08 | 8 | + | 37.5 3x  62.5 4x | SNP | Donnelly et al, 2014  Mixed tri- and tetrasomy |
| 24 | Htr8-11 | 8 | + | 91 | SNP | This work |
| 25 | Htr8-12 | 8 | + | 81 | SNP | This work |
| 26 | Htr8-13 | 8 | + | 90 | SNP | This work |
| 27 | Htr8-14 | 8 | + | 92 | SNP | This work |
| 28 | Htr8-15 | 8 | + | 79 | SNP | This work |
| 29 | Htr8-16 | 8 | + | 84 | SNP | This work |
| 30 | Htr8-19 | 8 | + | n/a | SNP | This work |
| 31 | Htr8-26 | 8 | + | n/a | SNP | This work |
| 32 | Htr8-27 | 8 | + | n/a | SNP | This work |
| 33 | Htr13-02 | 13 | - |  | SMASH | Domingues et al, 2017 |
| 34 | Htr13-03 | 13 | - |  | SMASH,WGS | Domingues et al, 2017 |
| 35 | Htr18-01 | 18 | - |  | SMASH | Domingues et al, 2017 |
| 36 | Htr18-02 | 18 | - |  | SMASH, WGS | Domingues et al, 2017 |
| 37 | Htr21-01 | 21 | - |  | SMASH | Domingues et al, 2017 |
| 38 | Htr21-03 | 21 | - |  | SMASH | Domingues et al, 2017 |
|  | **RPE1-derived cell lines** | | | | | |
|  | **Name** | **Chr.** | **H2B-GFP** | **% cells with WCA** | **Analysis** | **Comments** |
|  | RPE1 | - | - | 0 |  | Kind gift from Steven Taylor |
|  | RPE1 H2B-GFP | - | + | 0 |  | Kind gift from Steven Taylor |
| 1 | Rtr3-01 | 3 | - | 83 | SNP | Stingele et al, 2012, with a gain of 12 |
| 2 | Rtr3-02 | 3 | + | 100 | SNP, SMASH | Passerini et al, 2016, with a gain of 12 |
| 3 | Rtr3-03 | 3 | + | n/a | SNP | Passerini et al, 2016 |
| 4 | Rtr5-03 | 5 | + | 92 | SNP, Smash | Stingele et al, 2012, with a gain of 12 |
| 5 | Rtr5-07 | 5 | + | 100 | SNP | Passerini et al, 2016, with a gain of 12 |
| 6 | Rtr7-01 | 7 | + |  | SNP, WGS | Duerrbaum et al, 2018 |
| 7 | Rtr8-01 | 8 | - | 94 | SNP | This work |
| 8 | Rtr8-02 | 8 | - | 100 | SNP | This work |
| 9 | Rtr8-03 | 8 | - | 100 | SNP | This work |
| 10 | Rtr8-04 | 8 | - | 80 | SNP | This work |
| 11 | Rtr21-01 | 21 | - | n/a | SNP | Stingele et al, 2012 |
| 12 | Rtr21-02 | 21 | + | 90 | SNP | Stingele et al, 2012 |
| 13 | Rtr21-03 | 21 | + | 92 | SNP, SMASH | Stingele et al, 2012 |

Supplementary Table 1

Kneissig et al
