# Supplementary material for: Micronuclei-based model system reveals functional consequences of chromothripsis in human cells
Source: eLife. 2019 Nov 28;8:e50292. doi: 10.7554/eLife.50292 (PMC6910827; doi:10.7554/eLife.50292)
Supplement: Supplementary file 3. [file elife-50292-supp3.docx]

**Supplementary File 3 |** The number and percentage of informative SNPs per chromosomal region used for determination of the chromosome affected by unique CNAs; regions are determined per clone, by copy number. Legend is as follows:

1. Total number of SNPs in the specified region.
2. Number of SNPs in the specified region that is homozygous (B-allele frequency (BAF) <0.05 or >0.95) in the parental (par.) cell line.
3. Percentage of total SNPs that is homozygous (BAF <0.05 or >0.95) in the parental cell line.
4. Number of SNPs that showed a shift in B- allele frequency (BAF) to >0.15 and <0.85 after chromosome transfer, indicating the presence of the transferred chromosome for the specific SNP.
5. Percentage of SNPs homozygous in the parental cell line that showed a shift in B- allele frequency (BAF) to >0.15 and <0.85 after chromosome transfer, indicating the presence of the transferred chromosome for the specific SNP.
6. Percentage of the total number of SNPs in the specified region that showed a shift in B- allele frequency (BAF) to >0.15 and <0.85 after chromosome transfer, indicating the presence of the transferred chromosome for the specific SNP.

*This region is part of a large LOH region on chromosome 5 constitutionally present in the HCT116 cells used in this study.

| **Cell line** | **Chr.** | **Start** | **End** | **Event/copy number** | **SNPs in region^a^** | **Homozygous in par. cell line^b^** | **Homozygous in**  **par. cell line (%)^c^** | | **SNPs with BAF**  **shift^d^** | **SNPs with BAF shift (% of homozygous SNPs)^e^** | | **SNPs with BAF**  **shift (% of total)^f^** | |
| --- | --- | --- | --- | --- | --- | --- | --- | --- | --- | --- | --- | --- | --- |
| Htr3-2 | 3 | 1 | 122708761 | Normal | 10539 | 8630 | 81,9% | | 2766 | 32,1% | | 26,2% | |
| Htr3-2 | 3 | 122708761 | 198022430 | Deletion | 7096 | 4913 | 69,2% | | 209 | 4,3% | | 2,9% | |
| Htr5-4 | 5 | 1 | 63498708 | Normal | 8031 | 7509* | 93,5%* | | 2242 | 29,9% | | 27,9% | |
| Htr5-4 | 5 | 63498708 | 105185661 | Duplication | 2575 | 1702 | 66,1% | | 520 | 30,6% | | 20,1% | |
| Htr5-4 | 5 | 105389509 | 180915260 | Deletion | 7096 | 4925 | 69,4% | | 58 | 1,2% | | 0,8% | |
| Htr5-7 | 5 | 1 | 48719715 | Normal | 6451 | 6433* | 99,7%* | | 1980 | 30,8% | | 30,7% | |
| Htr5-7 | 5 | 48719715 | 180915260 | Deletion | 11257 | 7706 | 68,5% | | 2215 | 28,7% | | 19,7% | |
| Hte5-1 | 5 | 0 | 1241694 | Duplication | 104 | 104* | 100,0%* | | 25 | 24,0% | | 24,0% | |
| Hte5-1 | 5 | 1241695 | 7535101 | Deletion | 1052 | 1051* | 99,9%* | | 0 | 0,0% | | 0,0% | |
| Hte5-1 | 5 | 7535252 | 14619267 | Duplication | 955 | 954* | 99,9%* | | 349 | 36,6% | | 36,5% | |
| Hte5-1 | 5 | 14619843 | 14815619 | Deletion | 33 | 33* | 100,0%* | | 0 | 0,0% | | 0,0% | |
| Hte5-1 | 5 | 14815873 | 27183235 | Duplication | 1692 | 1686* | 99,6%* | | 395 | 23,4% | | 23,3% | |
| Hte5-1 | 5 | 27183236 | 32387298 | Deletion | 800 | 796* | 99,5%* | | 1 | 0,1% | | 0,1% | |
| Hte5-1 | 5 | 32387299 | 55284830 | Duplication | 2582 | 2368* | 91,7%* | | 687 | 29,0% | | 26,6% | |
| Hte5-1 | 5 | 55284831 | 56046900 | Deletion | 137 | 94 | 68,6% | | 0 | 0,0% | | 0,0% | |
| Hte5-1 | 5 | 56046901 | 60212685 | Duplication | 514 | 321 | 62,5% | | 57 | 17,8% | | 11,1% | |
| Hte5-1 | 5 | 60212750 | 73891689 | Deletion | 866 | 582 | 67,2% | | 1 | 0,2% | | 0,2% | |
| Hte5-1 | 5 | 73891980 | 93360824 | Duplication | 1306 | 868 | 66,5% | | 262 | 30,2% | | 20,1% | |
| Hte5-1 | 5 | 93360826 | 102652951 | Deletion | 492 | 315 | 64,0% | | 0 | 0,0% | | 0,0% | |
| Hte5-1 | 5 | 102767505 | 114632158 | Duplication | 954 | 654 | 68,6% | | 175 | 26,8% | | 18,3% | |
| Hte5-1 | 5 | 114632159 | 115123333 | Deletion | 45 | 26 | 57,8% | | 0 | 0,0% | | 0,0% | |
| Hte5-1 | 5 | 115123659 | 134192694 | Duplication | 1742 | 1204 | | 69,1% | 331 | | 27,5% | | 19,0% |
| Hte5-1 | 5 | 134192695 | 139421205 | Deletion | 306 | 212 | | 69,3% | 6 | | 2,8% | | 2,0% |
| Hte5-1 | 5 | 139421589 | 149896059 | Duplication | 774 | 515 | | 66,5% | 142 | | 27,6% | | 18,3% |
| Hte5-1 | 5 | 149896060 | 159594597 | Deletion | 766 | 545 | | 71,1% | 1 | | 0,2% | | 0,1% |
| Hte5-1 | 5 | 159594598 | 171429333 | Duplication | 1299 | 918 | | 70,7% | 296 | | 32,2% | | 22,8% |
| Hte5-1 | 5 | 171429334 | 172064649 | Deletion | 90 | 67 | | 74,4% | 0 | | 0,0% | | 0,0% |
| Hte5-1 | 5 | 172064659 | 175092845 | Duplication | 502 | 344 | | 68,5% | 101 | | 29,4% | | 20,1% |
| Hte5-1 | 5 | 175092846 | 175659799 | Deletion | 42 | 32 | | 76,2% | 7 | | 21,9% | | 16,7% |
| Hte5-1 | 5 | 175659892 | 176872016 | Duplication | 159 | 135 | | 84,9% | 33 | | 24,4% | | 20,8% |
| Hte5-1 | 5 | 176872017 | 178162354 | Deletion | 169 | 113 | | 66,9% | 0 | | 0,0% | | 0,0% |
| Hte5-1 | 5 | 178162446 | 180915260 | Duplication | 434 | 292 | | 67,3% | 70 | | 24,0% | | 16,1% |
| Htr8-1 | 8 | 1 | 22876657 | Deletion | 3494 | 2415 | | 69,1% | 0 | | 0,0% | | 0,0% |
| Htr8-1 | 8 | 22876657 | 146364022 | Normal | 12285 | 8525 | | 69,4% | 2014 | | 23,6% | | 16,4% |
| Htr8-2 | 8 | 1 | 41499358 | Deletion | 4938 | 3361 | | 68,1% | 1 | | 0,0% | | 0,0% |
| Htr8-2 | 8 | 41499358 | 62256246 | Normal | 1347 | 1009 | | 74,9% | 235 | | 23,3% | | 17,4% |
| Htr8-2 | 8 | 62256246 | 109010274 | Deletion | 4448 | 3061 | | 68,8% | 10 | | 0,3% | | 0,2% |
| Htr8-2 | 8 | 109010274 | 128388046 | Normal | 2416 | 1663 | | 68,8% | 335 | | 20,1% | | 13,9% |
| Htr8-2 | 8 | 128388046 | 146364022 | Deletion | 2630 | 1846 | | 70,2% | 3 | | 0,2% | | 0,1% |
| Htr8-5 | 8 | 1 | 89962337 | Normal | 7358 | 5022 | | 68,3% | 1531 | | 30,5% | | 20,8% |
| Htr8-5 | 8 | 89962337 | 139519958 | Duplication | 4377 | 2860 | | 65,3% | 796 | | 27,8% | | 18,2% |
| Htr8-5 | 8 | 139519958 | 146364022 | Normal | 646 | 425 | | 65,8% | 122 | | 28,7% | | 18,9% |
| Htr8-7 | 8 | 1 | 12060604 | Duplication | 1791 | 1233 | | 68,8% | 407 | | 33,0% | | 22,7% |
| Htr8-7 | 8 | 12060604 | 64766100 | Normal | 3887 | 2620 | | 67,4% | 782 | | 29,8% | | 20,1% |
| Htr8-7 | 8 | 64766100 | 71650441 | Deletion | 435 | 310 | | 71,3% | 1 | | 0,3% | | 0,2% |
| Htr8-7 | 8 | 71650441 | 116689545 | Normal | 3208 | 2084 | | 65,0% | 536 | | 25,7% | | 16,7% |
| Htr8-7 | 8 | 116689545 | 146364022 | Deletion | 3060 | 2060 | | 67,3% | 22 | | 1,1% | | 0,7% |
