# Supplementary material for: Micronuclei-based model system reveals functional consequences of chromothripsis in human cells
Source: eLife. 2019 Nov 28;8:e50292. doi: 10.7554/eLife.50292 (PMC6910827; doi:10.7554/eLife.50292)
Supplement: Supplementary file 5. [file elife-50292-supp5.docx]

**Supplementary File 5 |** Overview of informative SNPs deduced from whole genome sequencing reads, allowing determination of the affected chromosome. Legend is as follows:

1. ID: Unique breakpoint ID corresponding to Supplementary File 4
2. Chromosomal position of the informative SNP, coordinates are in hg19
3. Parental (HCT116) and donor (transferred chromosome) allele
4. Discordant reads with the parental/donor SNP supporting the specific genomic rearrangement
5. Normal reads with the parental/donor SNP allele supporting the specific genomic rearrangement

| **Cell line** | **ID (a)** | **Chr** | **Pos (b)** | **Parental (c) Donor (c)** | | **Discordant parental reads ( d)** | **Discordant donor reads (d)** | **Normal parental reads ( e)** | **Normal donor**  **reads ( e)** | **Affected chromosome** |
| --- | --- | --- | --- | --- | --- | --- | --- | --- | --- | --- |
| Hte5-01 | 031 | 5 | 115741207 | G | A | 0 | 17 | 76 | 40 | Transferred |
| Htr8-05 | 068 | 8 | 1167389 | G | C | 0 | 14 | 53 | 42 | Transferred |
| Htr8-05 | 068 | 8 | 1167584 | G | C | 0 | 3 | 61 | 41 | Transferred |
| Htr8-05 | 077 | 8 | 64763945 | T | C | 0 | 3 | 47 | 41 | Transferred |
| Htr8-05 | 086 | 8 | 134961461 | C | A | 0 | 3 | 71 | 66 | Transferred |
| Htr8-05 | 087 | 8 | 134961461 | C | A | 0 | 7 | 71 | 66 | Transferred |
| Htr8-05 | 073 | 8 | 134961461 | C | A | 0 | 5 | 71 | 66 | Transferred |
| Htr8-07 | 093 | 8 | 12486605 | T | A | 0 | 14 | 42 | 19 | Transferred |
| Htr8-07 | 093 | 8 | 71660840 | T | C | 0 | 14 | 155 | 53 | Transferred |
